# Supplementary material for: Loss of N-terminal acetyltransferase A activity induces thermally unstable ribosomal proteins and increases their turnover in Saccharomyces cerevisiae
Source: Nat Commun. 2023 Jul 27;14:4517. doi: 10.1038/s41467-023-40224-x (PMC10374663; doi:10.1038/s41467-023-40224-x)
Supplement: Supplementary file 1 — Supplementary information [file 41467_2023_40224_MOESM1_ESM.pdf]

Loss of N-terminal acetyltransferase A activity induces thermally unstable ribosomal proteins and increases their turnover in *Saccharomyces cerevisiae*

Supplementary information

Supplementary Figures 1-10

File Name: Supplementary Fig. 1. Lack of NatA activity (*naa10Δ* cells) elicit phenotypic changes by down-regulation of ribosomal proteins and up-regulation of autophagy markers.

Supplementary Fig. 2. Dilution constant effect on protein half-lives.

Supplementary Fig. 3. NatA substrates in the *naa10Δ* strain proteome shows faster normalized degradation rates compared to the full yeast proteome.

Supplementary Fig. 4. Lack of *NAA10* selectively reduces Nt-acetylation of NatA substrates.

Supplementary Fig. 5. Yeast strains lacking NatA have a normal ribosomal RNA processing as compared with WT.

Supplementary Fig. 6. Isothermal shift assay (ITSA) on soluble and precipitated fractions confirms that ribosomal proteins are non-stable in NatA defective yeast cells.

Supplementary Fig. 7. Yeast *naa10Δ* cells have similar cell cycle distribution to WT but are 20 % larger in size.

Supplementary Fig. 8. Gating report for the flow cytometry performed in Figure S7.

Supplementary Fig. 9. Landscape of the NatA substrates within the thermostability and degradation space in NatA defective yeast cells.

Supplementary Fig. 10. Effect of Tom1 on NatA defective yeast cells.

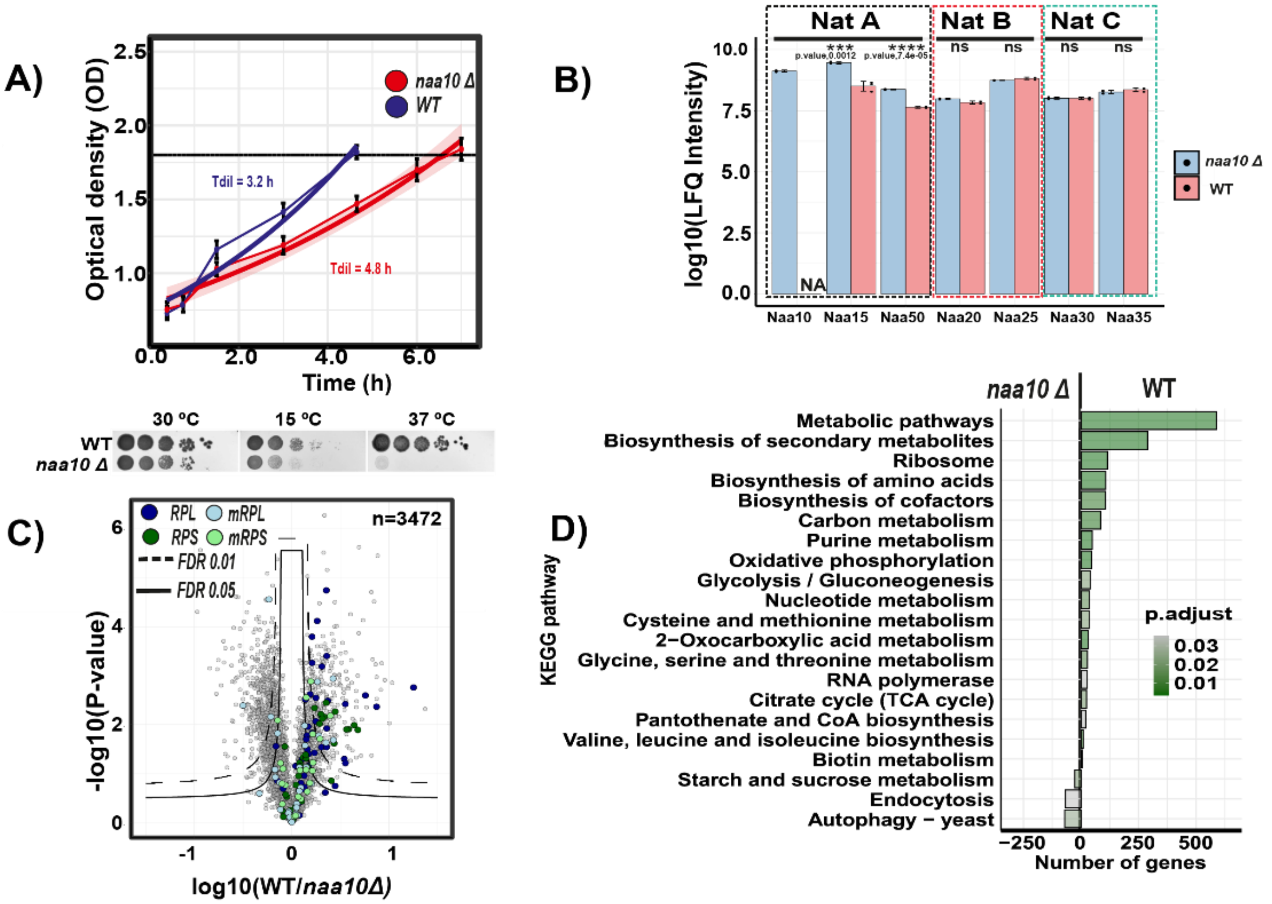

Supplementary Fig. 1. Lack of NatA activity (*naa10Δ* cells) elicit phenotypic changes by down-regulation of ribosomal proteins and up-regulation of autophagy markers. (A) Upper: WT and *naa10*Δ strain growth curves monitored by Optical density at a wavelength of 600 nm (OD). Cell cycle time is indicated for each condition (Tcc). Bottom: Phenotypic growth of *S. cerevisiae naa10Δ* at different temperatures. The indicated yeast strains were grown to early log phase and serial 1/10 dilutions containing the same number of cells were spotted on various media and imaged the six following days. 30 °C, incubated for 3 days on YPD at 30 °C; 15 °C, incubated for 6 days on YPD at 15 °C; 37 °C, incubated for 6 days on YPD at 37 °C. Results are representative of three independent experiments. **(B)** Relative abundance of the detected N-terminal acetyltransferase (NAT) subunits. Each identified protein was observed in at least two of the three biological replicates. Error bars represent standard deviation. Mean ± standard deviations are shown. Statistical significance was assessed using two-sided t-test, multiple test correction according to Benjamini-Hochberg, ns = P > 0.05, *P <=0.05, **P<=0.01, ***P<=0.001, **** P <= 0.0001 **(C)** Differential expression profiling of the WT and naa10Δ strains in a volcano plot. Significant regulated proteins at 1 % and 5% false discovery rate (FDR) are delimited by dashed and solid lines respectively (FDR controlled, two-sided t-test, randomizations = 250, s0 = 0.1) **(D)** GSEA-based KEGG pathway enriched analysis. *p-values* were calculated by two-sided permutation test and multiple hypothesis testing was FDR corrected. Significance threshold set at FDR > 0.05. Source data are provided as a Source Data file (SFig.1 A-C)

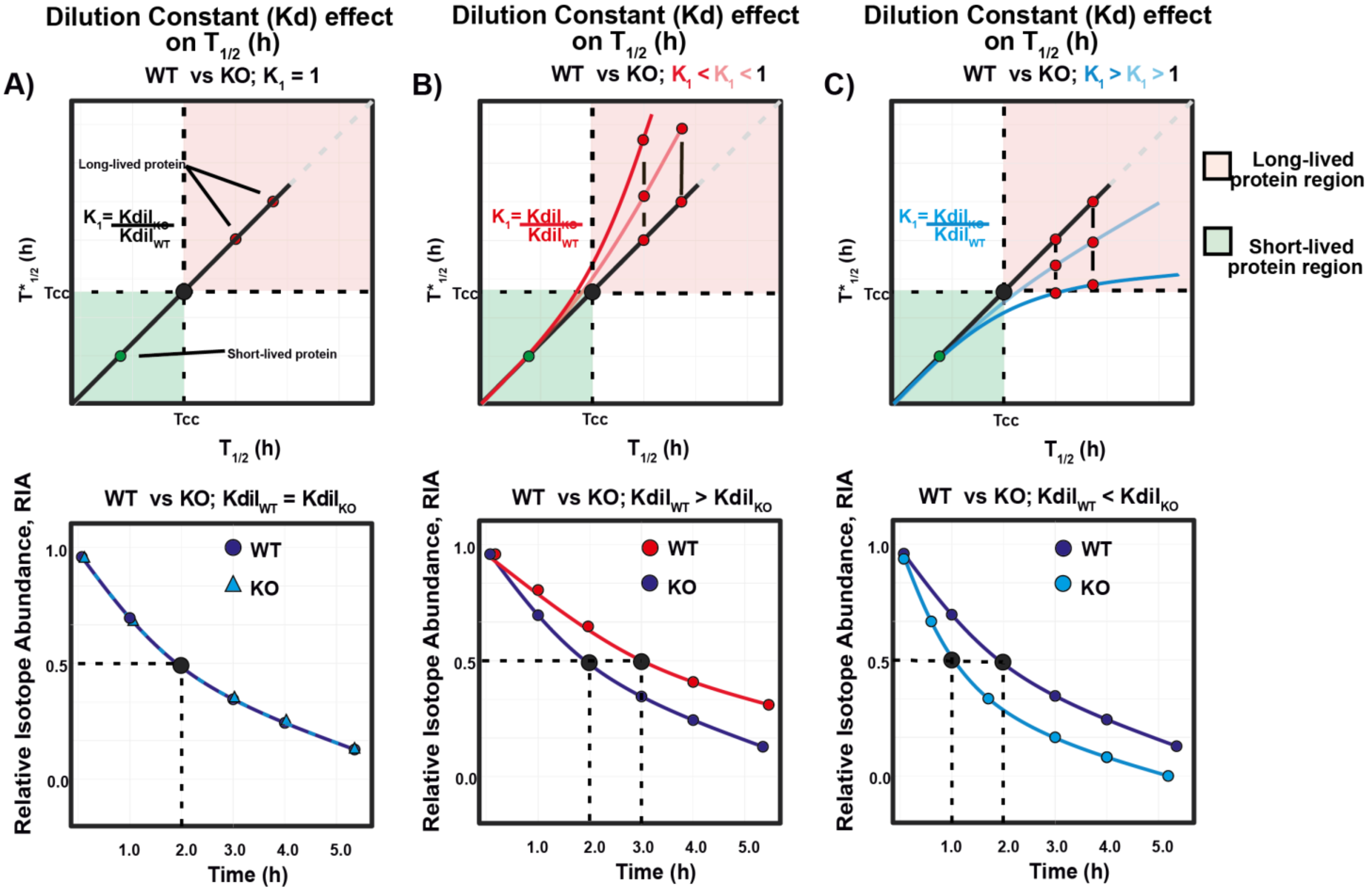

Supplementary Fig. 2. Dilution constant effect on protein half-lives. A) Upper: A comparison between WT half-lives (x axis) and calculated half-lives when the $K_1 = 1$ ($Kdil_{WT} = Kdil_{KO}$, y axis). Two regions are delimited (dashed line) as function of the cell cycle time (Black dot). Bottom: Diagram showing the time-domain RIA incorporation into naa10Δ and WT system proteins when the $Kdil_{WT} = Kdil_{KO}$, $K_1 = 1$. **B)** and **C)** Same as A, but when $Kdil_{WT} > Kdil_{KO}$, $K_1 < 1$ and $Kdil_{WT} < Kdil_{KO}$, $K_1 > 1$ respectively.

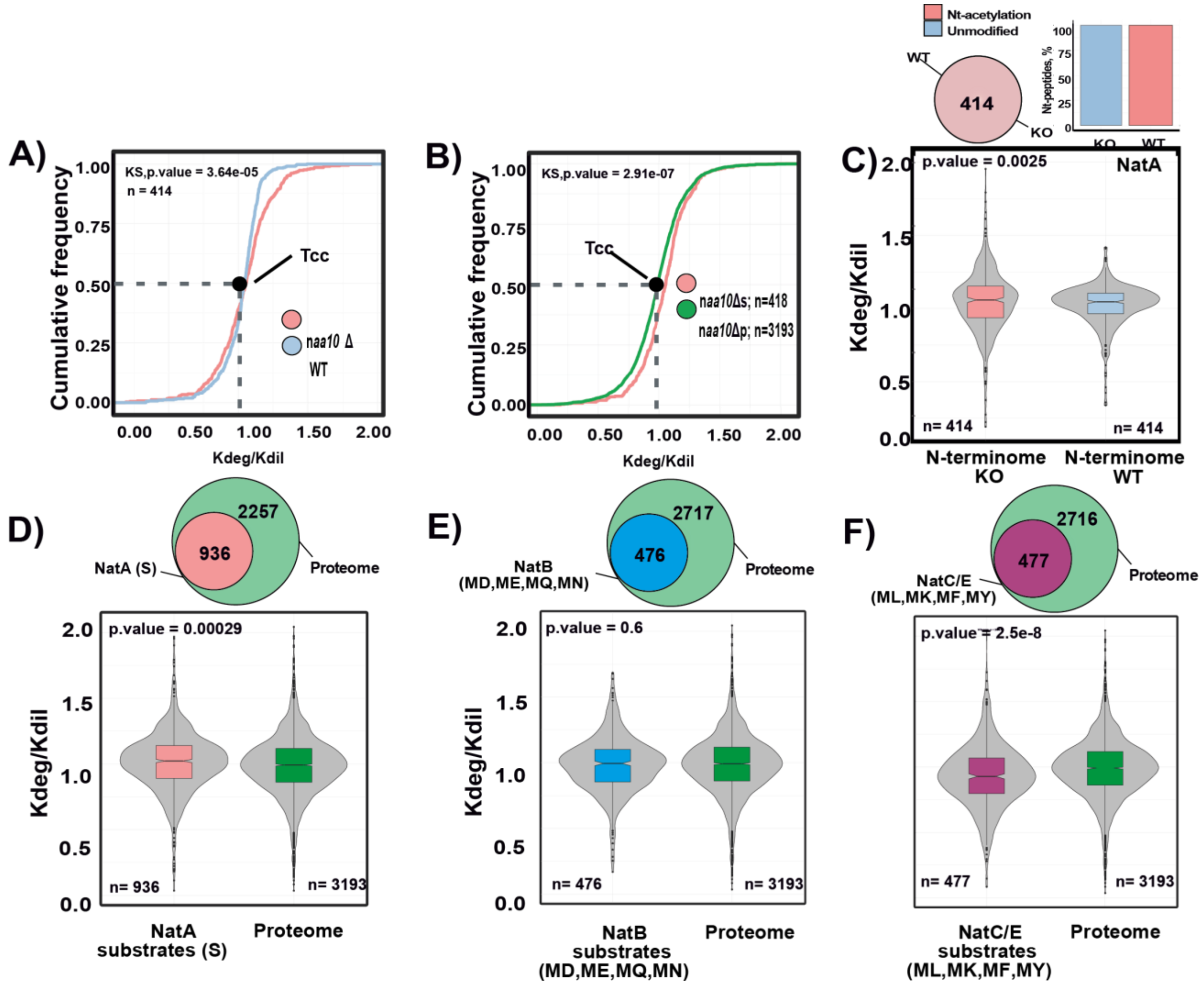

Supplementary Fig. 3. NatA substrates in the *naa10Δ* strain proteome shows faster normalized degradation rates compared to the full yeast proteome. (A) Cumulative frequency plot of the normalized turnover rate (Kdeg/Kdil) determined in *naa10Δ* and WT system of NatA substrates (WT, blue; *naa10Δ*, red). Two-sided Kolgomorov-Smirnov test, KS *P = -3.64 e-05*. **(B)** Cumulative frequency plot of the normalized turnover rate (Kdeg/Kdil) of NatA substrates and full proteome determined in *naa10Δ system* (*naa10Δ* proteome, blue; *naa10Δ* NatA substartes, red). Two-sided Kolgomorov-Smirnov test, KS *P = -3.64 e-05*. **(C)** Violin plot of normalized turnover rates of WT and *naa10Δ* NatA substrates compared to their corresponding N-terminome. Statistical significance was assessed using two-sided Wilcoxon test, multiple test correction according to Benjamini-Hochberg, ns = *P > 0.05, *P <=0.05, **P<=0.01, ***P<=0.001, **** P <= 0.0001*; box bounds correspond to quartiles of the distribution (center: median; limits: 1st and 3rd quartile; whiskers: +/− 1.5 IQR). Overlap between the N-terminome and proteome detected in the pSILAC, as well as the N-terminome acetylation status and NAT substrate class are shown on top (*n* = protein or peptide normalized turnover rates derived from at least two independent experiments per condition). **(D)** Same as C, but comparing NatA substrates with full proteome in *naa10Δ* condition. Only NatA substrates having a serine (S) in second amino acid of annotated protein sequences retrieved from the UniProt database were considered (*n* = protein normalized turnover rates derived from two independent experiments per condition). **(E-F)** Same as D but NatB (ME,MD,MQ and MN) and NatC/E (ML,MK,MF and MY) substrates were compared against the full proteome in *naa10Δ* condition respectively. Source data are provided as a Source Data file (SFig.3A-F).

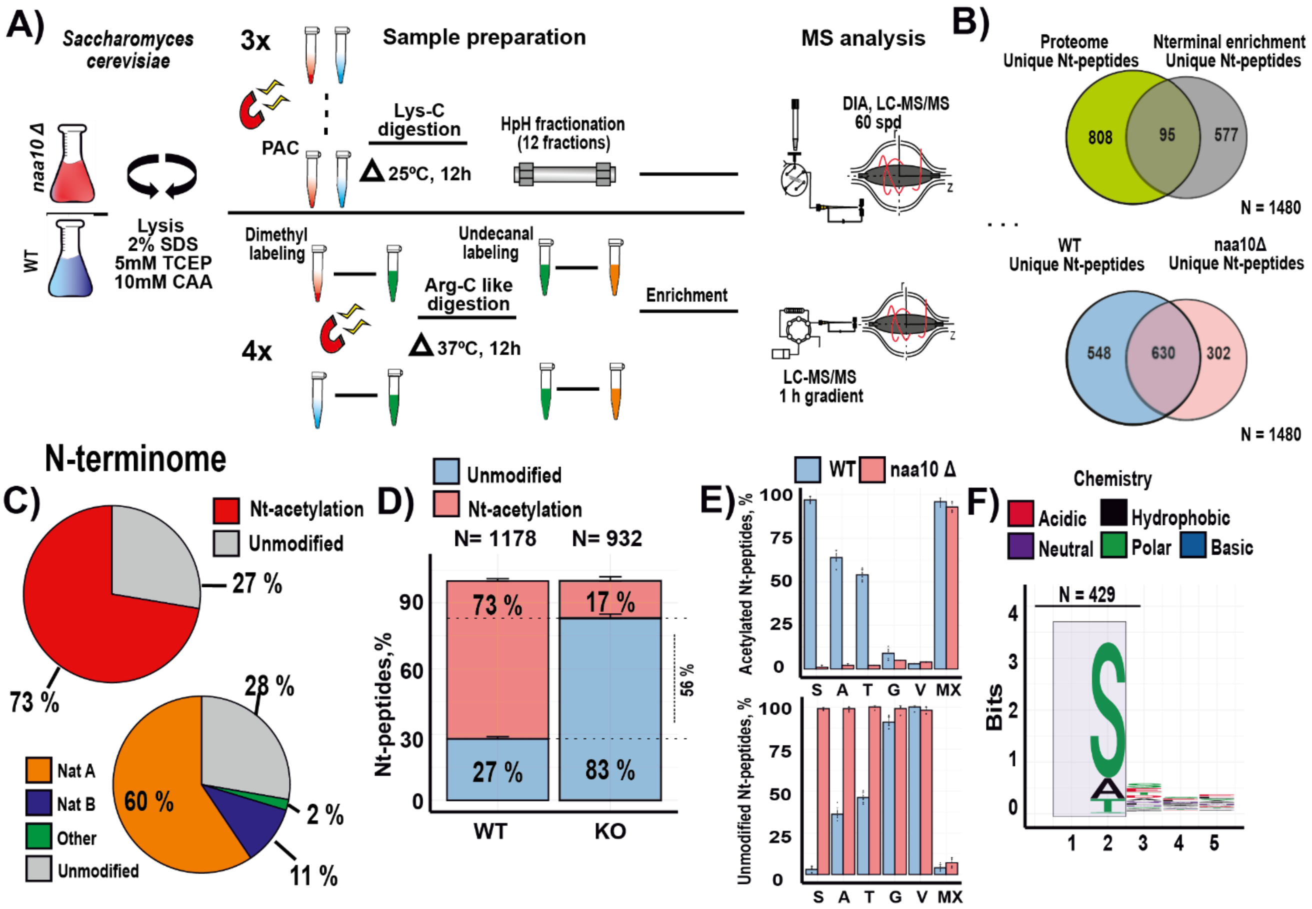

Supplementary Fig. 4. Lack of *NAA10* selectively reduces Nt-acetylation of NatA substrates. **(A)** Strategy implemented to determine the N-terminome of WT and *naa10*Δ strains. Yeast cells grown in mid log-phase were harvested and submitted to the indicated sample preparations. Unique Nt-peptides detected on HpH fractionation on Lys-C yeast digests and N-terminal enrichment were combined. n = 3 and n = 4 replicate cultures per condition, respectively. **(B)** Venn diagram depicting numbers of all unique Nt-peptides detected by biological condition and sample preparation. **(C)** Pie diagram depicting the NAT substrates classification and percentage of Nt-acetylation of the detected N-terminome. **(D)** Percentage of Nt-acetylation and unmodified N-termini detected in WT and *naa10Δ* yeast cells. Mean + standard deviation is shown. (*n* = 6, *n* = independent cultures per condition). **(E)** Upper: Percentage of Nt-acetylation found in proteins with N-terminal sequences matching the defined NatA and NatB substrate specificity. Mean + standard deviation is shown. Bottom: Percentage of unmodified Nt-peptides found in proteins with N-terminal sequences matching the defined NatA and NatB substrate specificity. Mean + standard deviation is shown. Ala-, Thr-, Ser-, Val-, and Gly- and X are in position +2. Met is in +1. X = Asn-, Asp-, Glu- and Gln at position 2+.(*n* = 6, *n* = independent cultures per condition). **(F)** Sequence logo summarizing the identity of the first five amino acids of the corresponding acetylated and unmodified peptides found in the WT and *naa10*Δ yeast N-terminomes. Source data are provided as a Source Data file (SFig.4 C-F)

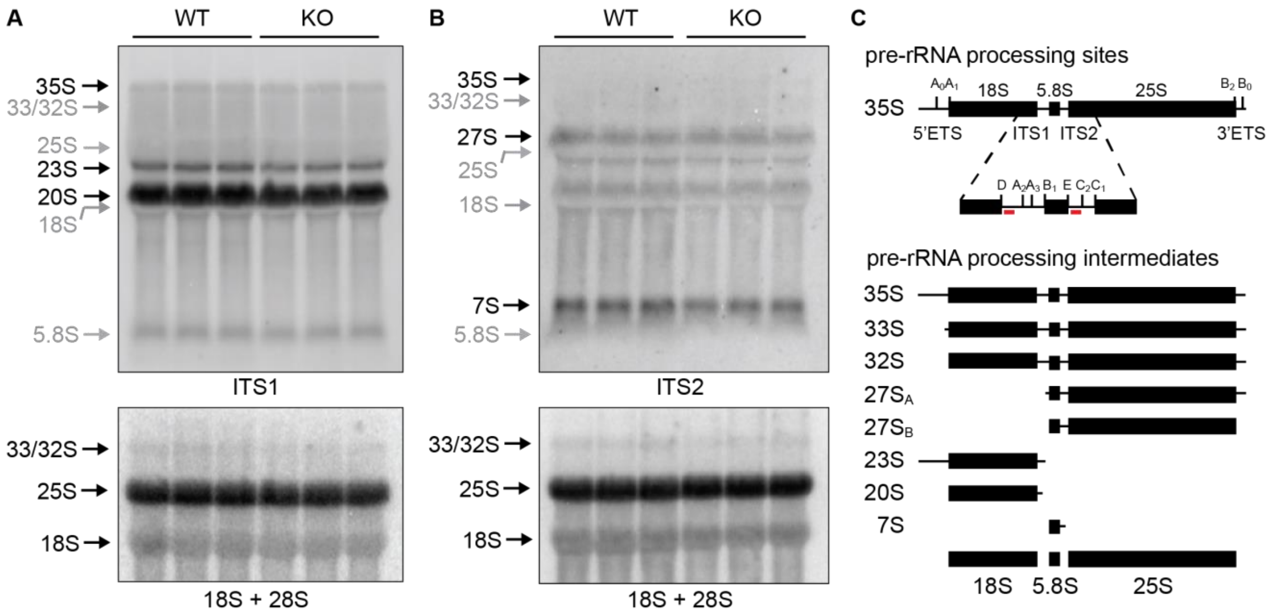

Supplementary Fig. 5. Yeast strains lacking NatA have a normal ribosomal RNA processing as compared with WT. (A) Northern blotting analysis of whole cell RNA isolated from WT and *naa10Δ* strains (KO), respectively (n=3, biological replicates) targeting ITS1 (upper panel) and the mature ribosomal subunits showing 18S and 25S (lower panel). **(B)** same as (A) except a probe targeting ITS2. **(C)** Simplified schematic illustration of the major pre-rRNA processing sites and intermediates. Black lines represent transcribed sequences including the external transcribed spacers (5 ETS and 3 ETS) and the internal transcribed spacers (ITS1 and ITS2) and black rectangles indicate the mature rRNAs (18S, 5.8S, and 25S). The hybridization location of probes targeting ITS1 and ITS2, respectively, are indicated (red bar) on the magnified view of the ITS1 and ITS2 regions within the 35S transcript.

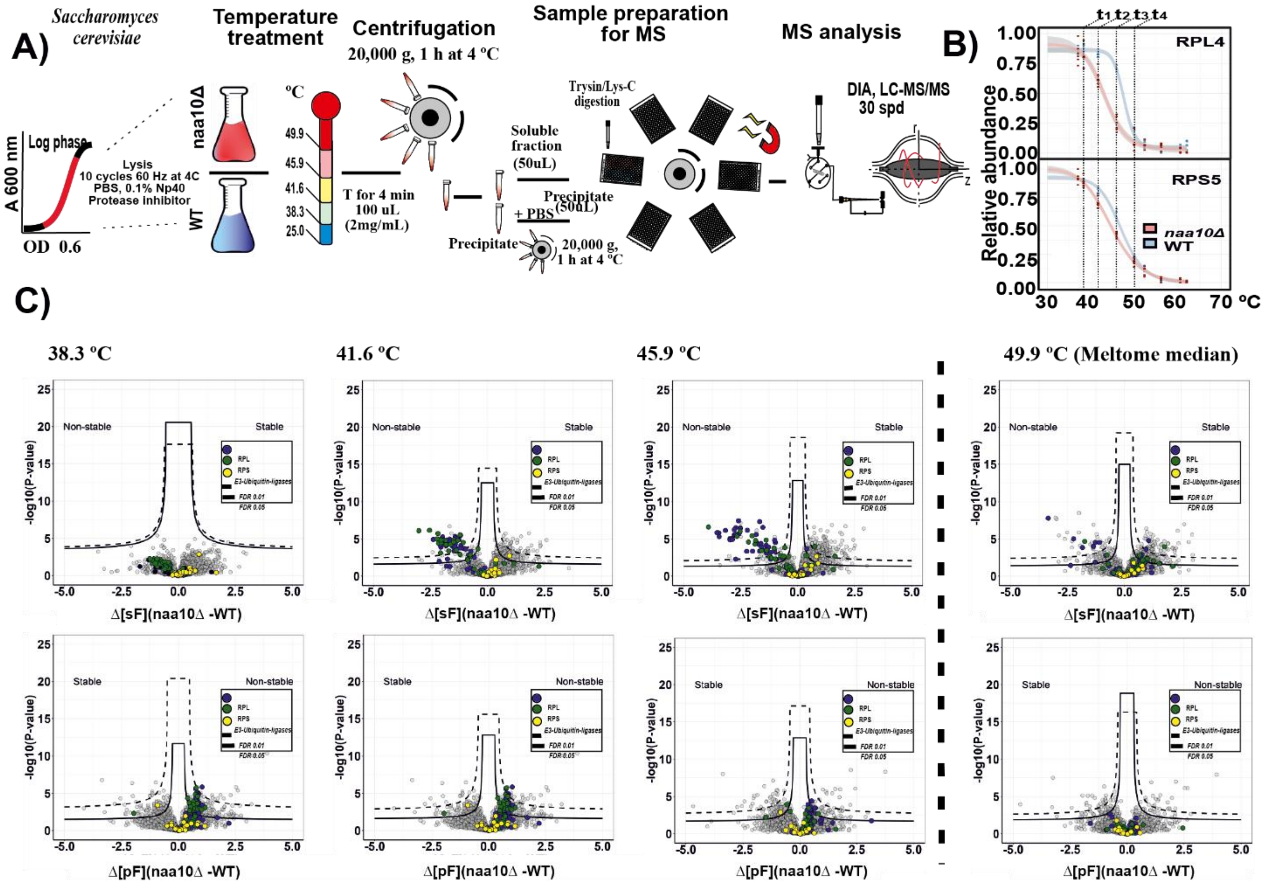

Supplementary Fig. 6. Isothermal shift assay (ITSA) on soluble and precipitated fractions confirms that ribosomal proteins are non-stable in NatA defective yeast cells. (A) Schematic representation of the implemented ITSA strategy. Yeast cells were harvested at mid log-phase and submitted to a temperature treatment (gradient of five temperatures). The supernatant and precipitated fractions were collected prior Lys-C/Trypsin digestion. Quantification was performed by using label-free intensities (LFQ; label-free quantitation). n = 6 replicate cultures per condition. **(B)** Representative melting curves of RPL4 and RPS5 are shown. The temperatures selected for the ITSA assay are shown on top. Error bars represent standard deviation. Mean ± standard deviations are shown. **(C)** Comparison of the WT and *naa10*Δ soluble and precipitated fractions in volcano plots to identify thermostability changes in the proteome. Significant regulated proteins at 1 % and 5% false discovery rate (FDR) are delimited by dashed and solid lines respectively (FDR controlled, two-sided t-test, randomizations = 250, s0 = 0.1). Source data are provided as a Source Data file (SFig.6 C)

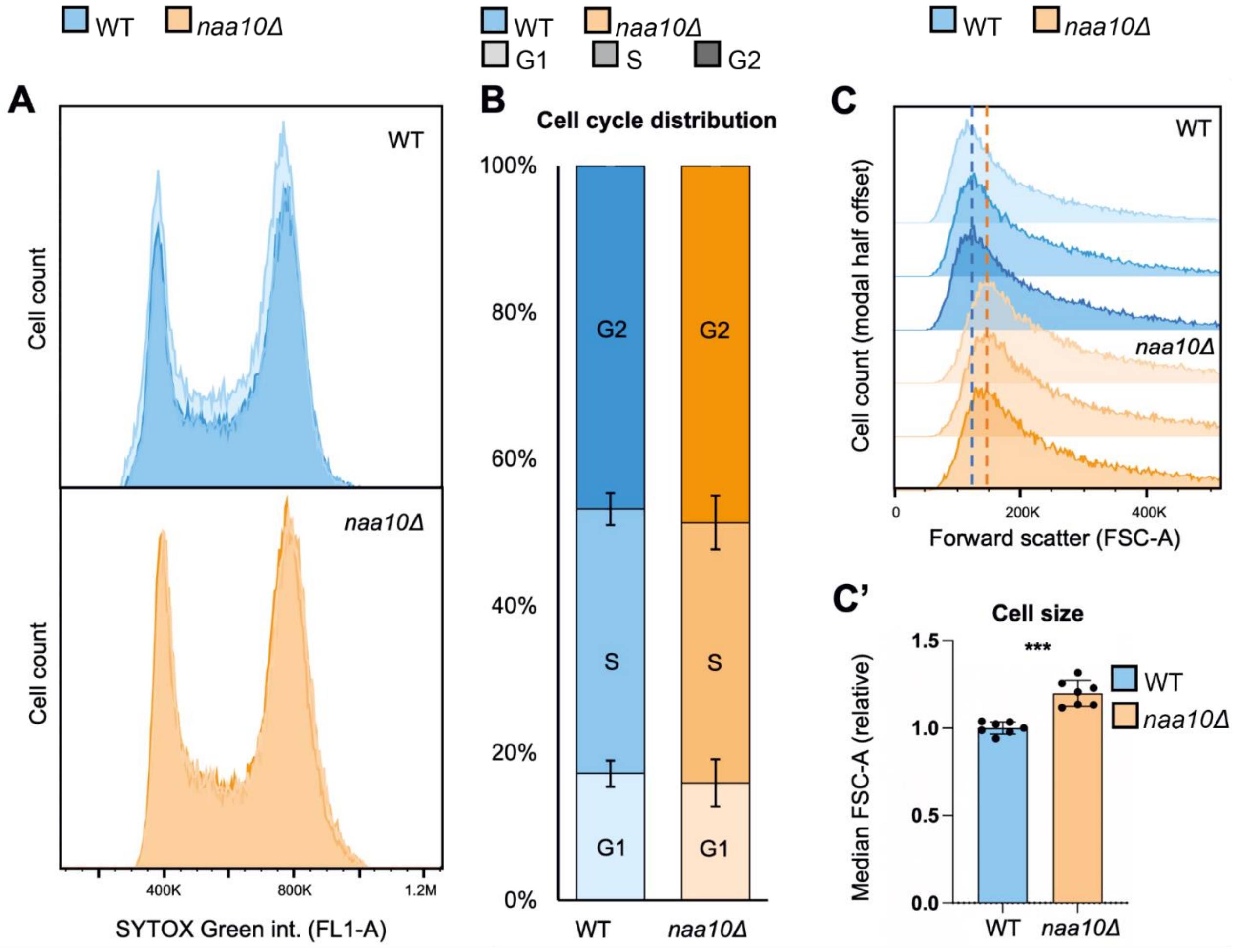

Supplementary Fig. 7. Yeast *naa10Δ* cells have similar cell cycle distribution to WT but are 20 % larger in size. Fig. S7. Yeast *naa10Δ* cells have similar cell cycle distribution to WT but are 20 % larger in size. Yeast cells were diluted from over-night preculture and grown in synthetic medium until OD 0.8-0.9, fixed and stained with SYTOX Green and analyzed by flow cytometry. (A) Cell cycle distribution profiles of WT vs *naa10Δ* cells. Shown is overlay of three replicates from one of three independent experimental setups. (B) Percentage of cells in G1, S or G2 phase from three replicate experimental setups with seven replicate samples in total. Data are presented as mean +/− s.d. Error bars show standard deviation. Differences among WT vs *naa10Δ* cells was analyzed for all stages using two-tailed t-test with unequal variance (G1, p=0.4; S, p=0.8; G2, p=0.2). (C) Corresponding forward scatter histogram plot for data in A. Dashed lines indicate a right-shift for the *naa10Δ* peak vs WT. C'. Median FSC-A value from three replicate experimental setups with seven replicate samples in total. *** indicate p=0.00017 using two-tailed t-test with unequal variance. Error bars represent standard deviation. Mean ± standard deviations are shown.

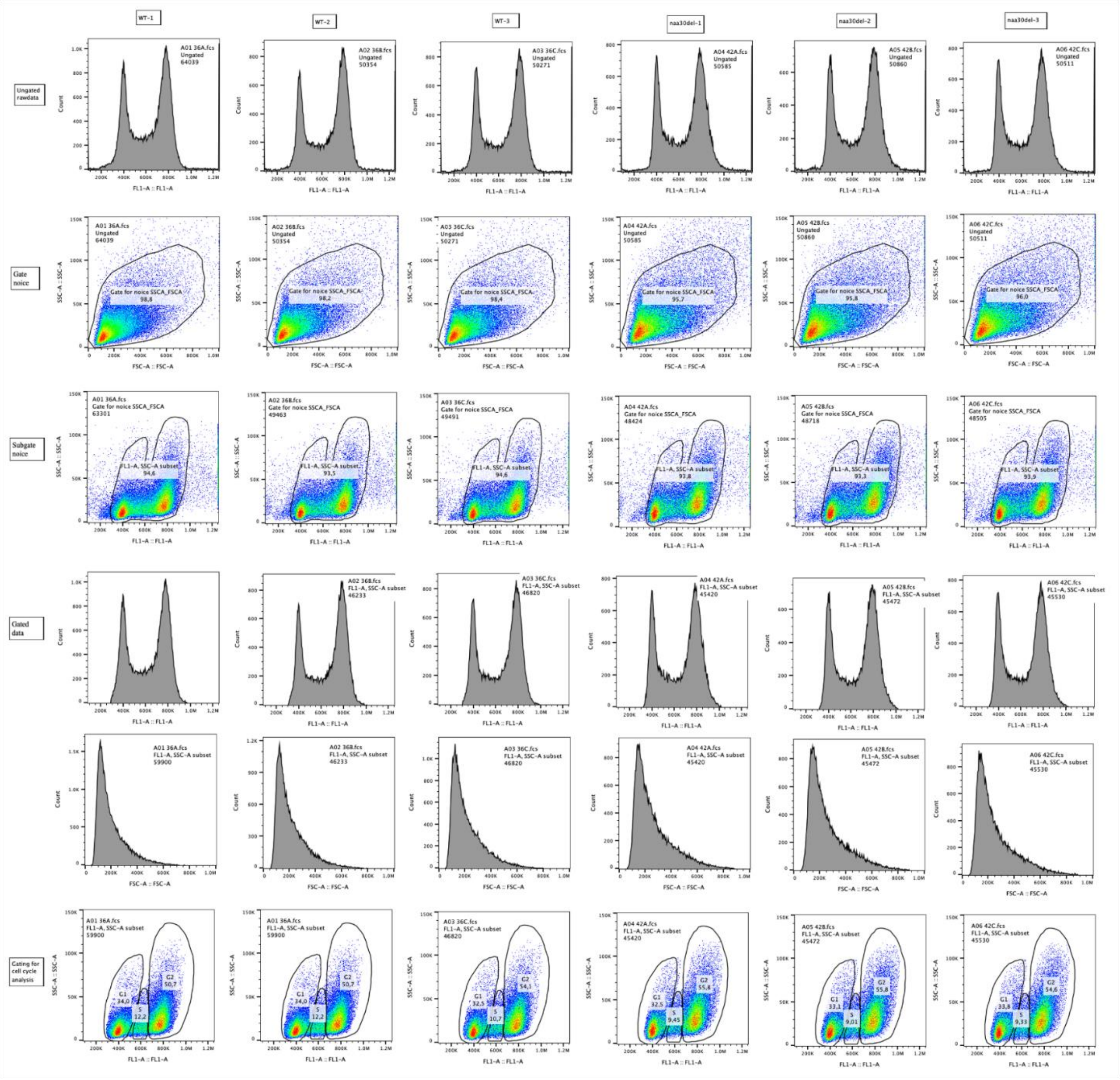

Supplementary Fig. 8. Gating report for the flow cytometry performed in Figure S7. Graphics showing the sequential gating strategies in the flow analysis. Three technical replicates from one experimental setup are shown (corresponding to data presented in Fig. S7A and C-C'). The last row shows the additional gating used for the cell cycle analysis (presented in Fig. S7B). The gating report is from one experimental setup and the same gating strategy was applied for all three independent experiments.

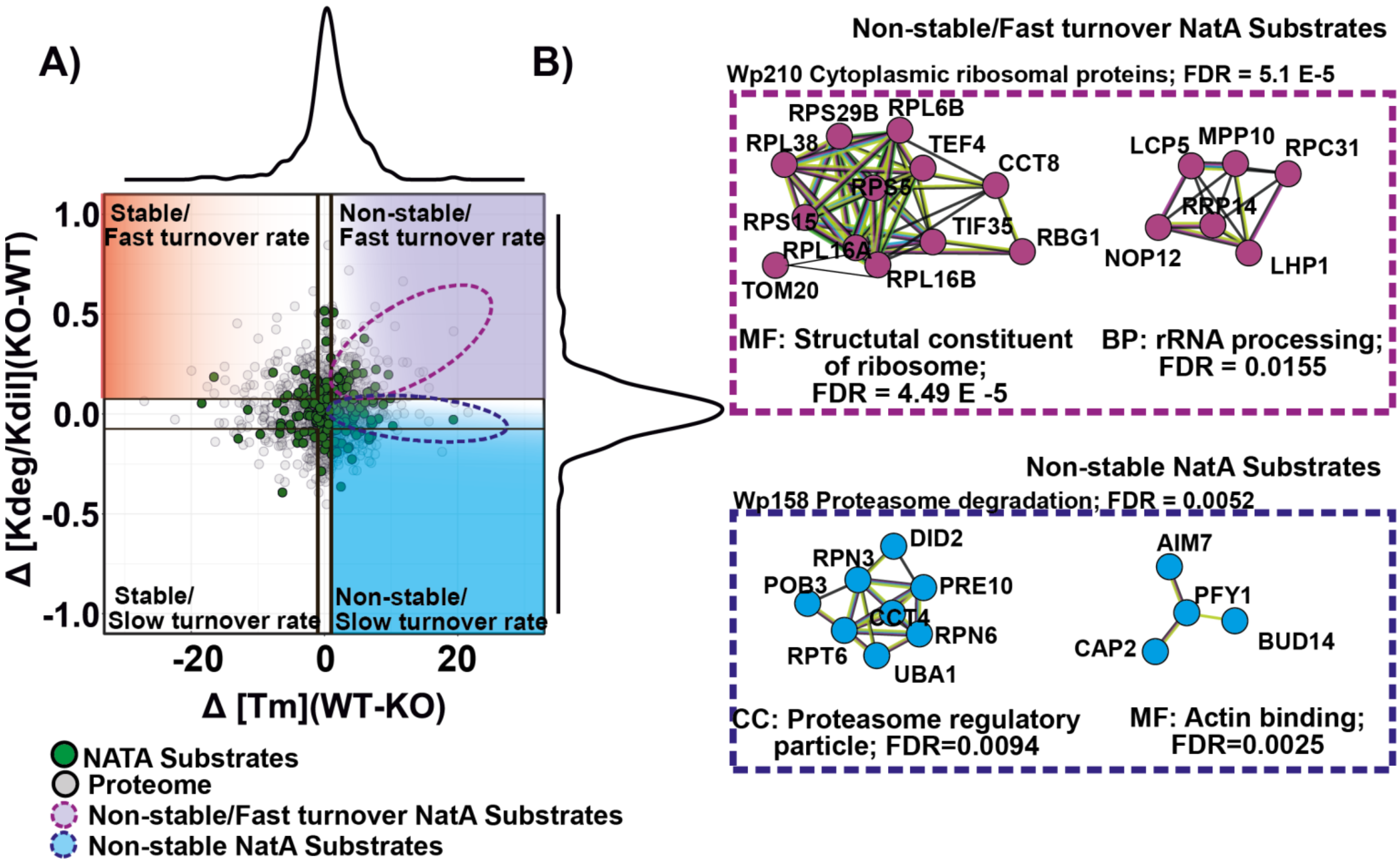

Supplementary Fig. 9. Landscape of the NatA substrates within the thermostability and degradation space in NatA defective yeast cells. (A) Scatterplot representing the overlap between protein thermostability and degradation. Black lines delineate the minimal fold change (s0, 0.1) in each dimension. Melting temperature and degradation difference distribution are shown in the top and right plot border respectively. Proteome and NatA substrates are colored in gray and green respectively. **(B)** String networks of the significantly enriched GO terms (BP: Biological Process; MF: Molecular Function and CC: Cellular component) within the Non-stable (Blue) and the Non-stable/Fast turnover space NatA substrates (Violet) are shown. Source data are provided as a Source Data file (SFig.9 A)

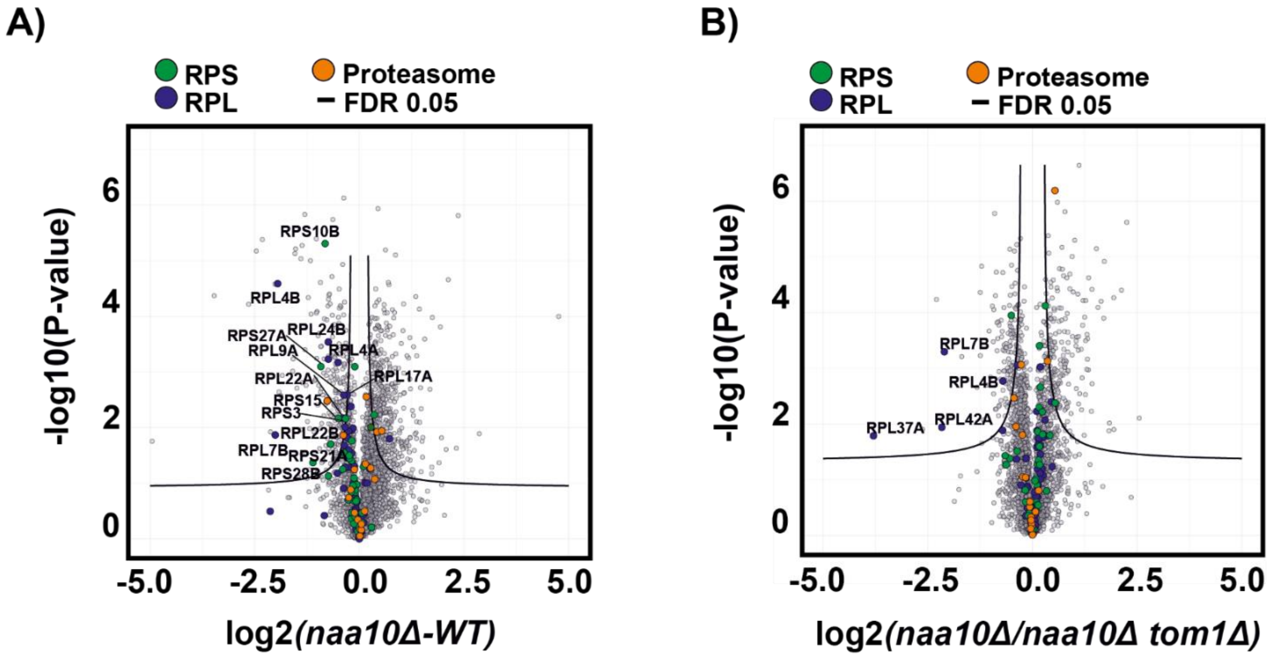

Supplementary Fig. 10. Effect of Tom1 on NatA defective yeast cells.
(A) Volcano plot displaying the log2 fold change against the t test–derived −log10 statistical P value for all proteins differentially expressed between WT and *naa10*Δ strains. **(B)** Same as A, but comparing the *naa10*Δ and *naa10*Δ *tom1*Δ strain proteomes. Significant regulated proteins at 1 % and 5% false discovery rate (FDR) are delimited by dashed and solid lines respectively (FDR controlled, two-sided *t*-test, randomizations = 250, s0 = 0.1). n = 3 replicate cultures per condition. Source data are provided as a Source Data file (SFig.10 A,B)
