# Supplementary material for: Loss of N-terminal acetyltransferase A activity induces thermally unstable ribosomal proteins and increases their turnover in Saccharomyces cerevisiae
Source: Nat Commun. 2023 Jul 27;14:4517. doi: 10.1038/s41467-023-40224-x (PMC10374663; doi:10.1038/s41467-023-40224-x)
Supplement: Supplementary file 2 — Additional supplementary files description [file 41467_2023_40224_MOESM2_ESM.pdf]

Description of Additional Supplementary Files

Loss of N-terminal acetyltransferase A activity induces thermally unstable ribosomal proteins and increases their turnover in Saccharomyces cerevisiae.

File Name: Supplementary Data 1

Description: This file includes the following tables.

Table S1.1. Contains all calculated degradation constants for each replicate

Table S1.2 Contains all calculated degradation constants for each replicate excluding replicate outside the Kdil MAD range

Table S1.3 Contains all calculated degradation constants for each replicate excluding replicate outside the Kdil MAD range

File Name: Supplementary Data 2

Description: This file includes the following tables.

Table S2.0 Averaged Kloss of unique Nterminal peptides from *Δnaa10* and WT strains

Table S2.1 Calculated Kloss of the corresponding unmodified and modified peptides from *Δnaa10* and WT strains. Propeptides, signal and transient peptides were removed

File Name: Supplementary Data 3

Description: This file includes the following tables.

Table S3.0 pSILAC Nat A substrates with Subcellular localization

File Name: Supplementary Data 4

Description: This file includes the following tables.

Table S4.0 All detected unique Nterminal peptides from *Δnaa10* and WT strains

Table S4.1 Overlapping detected unique Nterminal peptides between Δnaa10 and WT strains

File Name: Supplementary Data 5

Description: This file includes the following tables.

Table S5.0 Cytosolic ribosomal proteins from the large and small subunit classified by reported and detected Ntermini status (Nt-Ac, Nt-acetylated or Unmod, Unmodified)

File Name: Supplementary Data 6

Description: Table S6.0. Contains all calculated melting temperature for each replicate

File Name: Supplementary Data 7

Description: This file includes the following tables.

Table S7.0 Fast turnover ubiquitinated proteins

File Name: Supplementary Data 8

Description: This file contains R scripts for calculating melting curves and half-lives from TPP and pSILAC mass spectrometry data sets.
