# Supplementary material for: Loss of N-terminal acetyltransferase A activity induces thermally unstable ribosomal proteins and increases their turnover in Saccharomyces cerevisiae
Source: Nat Commun. 2023 Jul 27;14:4517. doi: 10.1038/s41467-023-40224-x (PMC10374663; doi:10.1038/s41467-023-40224-x)
Supplement: Supplementary file 11 — Reporting Summary [file 41467_2023_40224_MOESM11_ESM.pdf]

nature portfolio

Corresponding author(s): Jesper V. Olsen
Last updated by author(s): Jul 5, 2023

Reporting Summary

Nature Portfolio wishes to improve the reproducibility of the work that we publish. This form provides structure for consistency and transparency in reporting. For further information on Nature Portfolio policies, see our Editorial Policies and the Editorial Policy Checklist.

Statistics

For all statistical analyses, confirm that the following items are present in the figure legend, table legend, main text, or Methods section.

n/a	Confirmed	
☐	☒	The exact sample size (*n*) for each experimental group/condition, given as a discrete number and unit of measurement
☐	☒	A statement on whether measurements were taken from distinct samples or whether the same sample was measured repeatedly
☐	☒	The statistical test(s) used AND whether they are one- or two-sided<br>*Only common tests should be described solely by name; describe more complex techniques in the Methods section.*
☐	☒	A description of all covariates tested
☐	☒	A description of any assumptions or corrections, such as tests of normality and adjustment for multiple comparisons
☐	☒	A full description of the statistical parameters including central tendency (e.g. means) or other basic estimates (e.g. regression coefficient) AND variation (e.g. standard deviation) or associated estimates of uncertainty (e.g. confidence intervals)
☐	☒	For null hypothesis testing, the test statistic (e.g. *F*, *t*, *r*) with confidence intervals, effect sizes, degrees of freedom and *P* value noted<br>*Give P values as exact values whenever suitable.*
☒	☐	For Bayesian analysis, information on the choice of priors and Markov chain Monte Carlo settings
☒	☐	For hierarchical and complex designs, identification of the appropriate level for tests and full reporting of outcomes
☒	☐	Estimates of effect sizes (e.g. Cohen's *d*, Pearson's *r*), indicating how they were calculated

Our web collection on statistics for biologists contains articles on many of the points above.

Software and code

Policy information about availability of computer code

Data collection	Mass spectrometry data was acquired using the Orbitrap Exploris 480 MS (Thermo Fisher Scientific, Bremen, Germany) and the Orbitrap Q Exactive HF-X MS (Thermo Fisher Scientific, Bremen, Germany), using Xcalibur (tune version 3.0).
Data analysis	Proteomics data was analyzed using Spectronaut (v15) and MaxQuant (1.6.7.0) and further processed in R (v4.2.1), Prostar (v1.28.0) or Perseus (v1.6.7.0). Custom R code is provided as a Supplementary Data File.

For manuscripts utilizing custom algorithms or software that are central to the research but not yet described in published literature, software must be made available to editors and reviewers. We strongly encourage code deposition in a community repository (e.g. GitHub). See the Nature Portfolio guidelines for submitting code & software for further information.

Data

Policy information about availability of data

All manuscripts must include a data availability statement. This statement should provide the following information, where applicable:

- Accession codes, unique identifiers, or web links for publicly available datasets
- A description of any restrictions on data availability
- For clinical datasets or third party data, please ensure that the statement adheres to our policy

The Mass spectrometry-based proteomics data generated to determine pSILAC chase, TPP-DIA, ITSA, Deep proteome and ubiquitinome at steady-state analysis in this study have been deposited in the ProteomeXchange Consortium via PRIDE with the identifier PXD037510(https://proteomecentral.proteomexchange.org/cgi/GetDataset?ID=PXD037510). Processed pSILAC chase, TPP-DIA, ITSA, Deep proteome and ubiquitinome at steady-state analysis data are provided in Supplementary

Information/Source Data file. The Flow cytometry data generated in this study have been deposited on flowrepository.org repository under the identifier FR-FCM-Z6KA.
(http://flowrepository.org/id/RvFrQTWTEN1QtSMlScADYGOFAruldGnBwKBwIvQV8l3bgPuJK0HTt4J4Q0kX4l8E)
Yeast UniProt database was used for this study (https://www.uniprot.org/)

Research involving human participants, their data, or biological material

Policy information about studies with human participants or human data. See also policy information about sex, gender (identity/presentation), and sexual orientation and race, ethnicity and racism.

Reporting on sex and gender	Not relevant
Reporting on race, ethnicity, or other socially relevant groupings	Not relevant
Population characteristics	Not relevant
Recruitment	Not relevant
Ethics oversight	Not relevant

Note that full information on the approval of the study protocol must also be provided in the manuscript.

Field-specific reporting

Please select the one below that is the best fit for your research. If you are not sure, read the appropriate sections before making your selection.

☒ Life sciences ☐ Behavioural & social sciences ☐ Ecological, evolutionary & environmental sciences

For a reference copy of the document with all sections, see nature.com/documents/nr-reporting-summary-flat.pdf

Life sciences study design

All studies must disclose on these points even when the disclosure is negative.

Sample size	In each of the experiments, we analyzed 3 to 6 replicates. The sample size was determined considering that we utilized independent cultures for each replicate and condition. Furthermore, a sample size of three independent replicates is widely accepted within the scientific community.
Data exclusions	One replicate of the pSILAC experiment was excluded from the analysis due to being identified as an experimental outlier. This determination was made based on the calculated Kdil values, which fell outside the median absolute deviation (MAD) range.
Replication	All the experiments were performed using independent culture replicates as stated in the sample size section. All the replicates were processed in parallel. All the cases the replication was successful.
Randomization	As the experimental design of this study involve multiple conditions only the comparison between knockout (KO) and wild-type (WT) samples, and not multiple conditions and groups, the use of randomization is not relevant in this context.
Blinding	Since we performed a discovery-based proteomics study, the experimental design required the pre-knowledge of the treatment or condition. Therefore, conducting a blind study may not be as critical.

Reporting for specific materials, systems and methods

We require information from authors about some types of materials, experimental systems and methods used in many studies. Here, indicate whether each material, system or method listed is relevant to your study. If you are not sure if a list item applies to your research, read the appropriate section before selecting a response.

Materials & experimental systems

n/a	Involved in the study
[x]	[ ] Antibodies
[ ]	[x] Eukaryotic cell lines
[x]	[ ] Palaeontology and archaeology
[ ]	[x] Animals and other organisms
[x]	[ ] Clinical data
[x]	[ ] Dual use research of concern
[x]	[ ] Plants

Methods

n/a	Involved in the study
[x]	[ ] ChIP-seq
[ ]	[x] Flow cytometry
[x]	[ ] MRI-based neuroimaging

Eukaryotic cell lines

Policy information about cell lines and Sex and Gender in Research

Cell line source(s)	The S. cerevisiae strains used were S288C isogenic yeast strains (MATα) wild-type, BY4742 (Y10000, EUROSCARF; internal reference Arnesenlab yTA36); and the thereof modified naa10Δ, YHR013C-Δ::kanMX4 (Y10976, EUROSCARF; internal reference Arnesenlab yTA42).
Authentication	The S. cerevisiae strains used were S288C isogenic yeast strains (MATα) wild-type, BY4742 (Y10000, EUROSCARF; internal reference Arnesenlab yTA36); and the thereof modified naa10Δ, YHR013C-Δ::kanMX4 (Y10976, EUROSCARF; internal reference Arnesenlab yTA42).
Mycoplasma contamination	Cell lines not tested for mycoplasma
Commonly misidentified lines (See ICLAC register)	N/A

Animals and other research organisms

Policy information about studies involving animals; ARRIVE guidelines recommended for reporting animal research, and Sex and Gender in Research

Laboratory animals	N/A
Wild animals	N/A
Reporting on sex	N/A
Field-collected samples	N/A
Ethics oversight	N/A

Note that full information on the approval of the study protocol must also be provided in the manuscript.

Flow Cytometry

Plots

Confirm that:

- [x] The axis labels state the marker and fluorochrome used (e.g. CD4-FITC).
- [x] The axis scales are clearly visible. Include numbers along axes only for bottom left plot of group (a 'group' is an analysis of identical markers).
- [x] All plots are contour plots with outliers or pseudocolor plots.
- [x] A numerical value for number of cells or percentage (with statistics) is provided.

Methodology

Sample preparation	Yeast were grown in SC medium and harvested at the same conditions as for the other analyses, before fixation and staining with the DNA-binding dye SYTOX Green (Invitrogen™ #S7020), which has been shown to outperform propidium iodide providing more reliable stain with improved linearity between DNA content and fluorescence.At harvest, 1 ml culture was centrifuged at 4,000 xg for 10 min, before the cells were resuspended in 1.5 ml Milli-Q water. The cells were fixed by drop-wise addition of 3.5 ml 100 % ethanol at 1,400 rpm vortexing and incubated on a rotating wheel (15 rpm) overnight at 4C. The cells were washed in 1 ml Milli-Q water and incubated in 500l heat-treated RNase solution (Qiagen #19101) for 4 hrs at 37C. The cells were pelleted, treated with 200l pepsin protease solution (Roche #10108057001) for 15 min at 37C, before resuspension in 500 l 50 mM Tris, pH 7.5 and storage at 4C. 50l cell solution was mixed with 1 ml SYTOX Green solution in a dark microtube and then sonicated at 20 kHz for 5x 2 sec pulses on ice. The DNA content reported by the SYTOX

	Green signal intensity was measured using Accuri C6 (Flow cytometry core facility, Bergen) and the FL1 detector with a standard 530/30 band pass filter. The limit was set to 5,000 cells and fluidics speed was set to fast. Three independent experiments with seven replica cultures in total were run. Data was processed, analyzed and visualized using FlowJo. Cell cycle analysis was performed using the cell cycle tool in FlowJo applying the Watson (Pragmatic) model and equal range for C1 and C2, optimized for lowest possible RMSD. Cell cycle distribution % values were exported and statistical testing was performed using two-tailed t-test with unequal variance
Instrument	Accuri C6 (Flow cytometry core facility, Bergen)
Software	FlowJo
Cell population abundance	The flow experiment were set up to determine cell cycle stage of the yeast strains. G2 40 %, G1 and S 20%
Gating strategy	The gating strategy was performed using on the SSC-A vs FL1-A. Commonly used for gate cell populations based on their side scatter (SSC) and fluorescence intensity in the FL1 channel.

☒ Tick this box to confirm that a figure exemplifying the gating strategy is provided in the Supplementary Information.
